# Understanding tobacco industry pricing strategy and whether it undermines tobacco tax policy: the example of the UK cigarette market

**DOI:** 10.1111/add.12159

**Published:** 2013-04-16

**Authors:** Anna B Gilmore, Behrooz Tavakoly, Gordon Taylor, Howard Reed

**Affiliations:** 1 Department for Health, University of BathBath, UK; 2 Landman EconomicsColchester, UK

**Keywords:** Cigarette prices, cigarette taxation, tobacco industry, tobacco industry pricing strategy, tobacco tax policy

## Abstract

**Aims:**

Tobacco tax increases are the most effective means of reducing tobacco use and inequalities in smoking, but effectiveness depends on transnational tobacco company (TTC) pricing strategies, specifically whether TTCs overshift tax increases (increase prices on top of the tax increase) or undershift the taxes (absorb the tax increases so they are not passed onto consumers), about which little is known.

**Design:**

Review of literature on brand segmentation. Analysis of 1999–2009 data to explore the extent to which tax increases are shifted to consumers, if this differs by brand segment and whether cigarette price indices accurately reflect cigarette prices.

**Setting:**

UK.

**Participants:**

UK smokers.

**Measurements:**

Real cigarette prices, volumes and net-of-tax- revenue by price segment.

**Findings:**

TTCs categorise brands into four price segments: premium, economy, mid and ‘ultra-low price’ (ULP). TTCs have sold ULP brands since 2006; since then, their real price has remained virtually static and market share doubled. The price gap between premium and ULP brands is increasing because the industry differentially shifts tax increases between brand segments; while, on average, taxes are overshifted, taxes on ULP brands are not always fully passed onto consumers (being absorbed at the point each year when tobacco taxes increase). Price indices reflect the price of premium brands only and fail to detect these problems.

**Conclusions:**

Industry-initiated cigarette price changes in the UK appear timed to accentuate the price gap between premium and ULP brands. Increasing the prices of more expensive cigarettes on top of tobacco tax increases should benefit public health, but the growing price gap enables smokers to downtrade to cheaper tobacco products and may explain smoking-related inequalities. Governments must monitor cigarette prices by price segment and consider industry pricing strategies in setting tobacco tax policies.

## Introduction

Raising tobacco taxes and prices is one of the most effective means of reducing tobacco use, particularly in the young and the less well-off—who are known to be the most price sensitive 1–6. Price increases are also the most likely intervention to reduce inequalities in smoking 7,8. However, the effectiveness of tobacco tax policies depends on tobacco company pricing strategies. Tobacco companies can choose to absorb the tax increase so that it is not passed onto to consumers as an increase in price, thus undermining the effect of tobacco tax policy (a practice known as undershifting); to pass it onto consumers in full; or to increase prices on top of the tax increases (a practice known as overshifting and which increases both the effect of the tax increase and industry revenue). Yet, surprising little is known about tobacco industry pricing strategies 6 and no studies have yet addressed this issue in the UK 6.

This article therefore aims to examine tobacco industry pricing strategy in the UK. Specifically, it aims to identify how the tobacco industry segments its cigarettes by price, and then to examine how price, volume and revenue trends vary by price segment, the extent to which tobacco tax increases are shifted to consumers, whether this differs by price segment and whether commonly used price indices adequately measure cigarette price trends.

### Background: tax policy and structure

The parameters within which UK tobacco tax policy is set are determined by European Union (EU) legislation requiring member states to have a mixed tobacco excise structure with both proportional (ad valorem) and fixed (specific) elements. Until January 2011 excise taxes were based on the retail selling price of the price category most in demand, commonly known as the most popular price category (MPPC). Directive 2010/12/EU 9, which entered into effect on 1 January 2011, changes the reference point for calculating taxes from the MPPC to the weighted average retail selling price.

## Methods

### Literature review: price segmentation

The study was approved by the University of Bath Research Ethics Approval Committee for Health. A comprehensive review of the literature on brand segmentation and cigarette prices covering the period 1999 to 2009 was used to inform the allocation of brands to price segments. The review, which included the academic literature, market reports (Euromonitor, KeyNote and Mintel), industry analyst reports, tobacco manufacturer annual reports, and tobacco industry and retail journals (*Tobacco Journal International*; *Retail Newsagent*, *The Grocer*, *ProWholesaler* and *Talking Retail*) recorded carefully (i) the number of price segments identified and (ii) the names of each brand identified in a particular price segment at any point in time. More than 80 relevant articles or reports were identified, with only one coming from the academic literature 10. Full details are available elsewhere 11.

This review showed that most sources reported three or four price segments. In recent years, four segments have more consistently been reported. This is because, from approximately 2005, a new ‘ultra-low price’ (ULP) segment, which had not featured consistently before, began to be reported in the industry and retail literature 12–17 as the transnational tobacco companies (TTCs) began to acquire these brands from supermarkets, which had sold them for a number of years 10, and to launch their own ULP brands. For example, in January 2006 Imperial Tobacco acquired the Windsor Blue brand which had been sold by the Cooperative supermarket for 30 years 15,18 and in November 2008 launched brand-variants John Player Special (JPS) Silver, Blue and Menthol as a cut-price versions of its longstanding JPS brand 19.

On the basis of this literature review, cigarette brands were categorised into four price segments—premium, mid-price, economy and ULP. As TTCs only acquired or launched ULP brands from the mid-2000s onwards, price data for this segment were only available from 2006.

### Data

#### Price data

Brand-specific cigarette price data from 1999 to 2009 were obtained for packs of 20 cigarettes (or 19 cigarettes for brands sold only in 19s). No single source covering the same geographical area was available for the whole period. The main source of price data until October 2005, when it ceased publication, was *PriceChecker*, a supplement to the *Retail Newsagent* magazine from which data were extracted by hand. This gave recommended retail prices (RRP) for all major cigarette brands for the UK market (i.e. Great Britain and Northern Ireland wherein Northern Ireland accounts for just 3% of total volume 20). From November 2006 Nielsen started to publish cigarette sales data, including volume and price, for the Great Britain market (i.e. excluding Northern Ireland) and obtained, via scan, data inputs covering 87% of total sales 21.

Comparisons between these two datasets, supermarket retail prices and manufacturers’ RRPs showed that manufacturers’ RRP and Nielsen data were almost identical, indicating that our two data sources (*PriceChecker* and Nielsen) could be relied on to provide comparable price data over time. The expense of Nielsen data precluded our ability to collate monthly data. Data were therefore obtained for May/June and November/December each year (other than in 2005 when October data were used in place of November/December as October was the last published edition of *PriceChecker*).

#### Volume market share data

Data on market share by volume were obtained from two sources: Nielsen from November 2006 until November 2009 as detailed above 22 and the General Household Survey (GHS), now known as the General Lifestyle Survey 23, for the period 2001 to 2008 (2008 being the most recent survey available at the time this work was undertaken). The GHS is an annual survey designed to be representative of the population of the UK. Each year, one question, posed only to smokers of manufactured cigarettes aged 16 years plus, asks which brand of cigarettes they smoke. This question, combined with the number of sticks smoked per week by each smoker, was used to calculate market share by brand and price segment.

### Analysis

#### Trends in cigarette prices, volumes and revenue by price segment

Weighted average prices for a pack of 20 cigarettes were calculated for the period 2001 to 2009 by price segment using GHS market share data for the period 2001 to 2005 and Nielsen market share data from 2006 to 2009. Real prices were then calculated using both the Retail Price Index (RPI; the UK government's preferred measure of inflation during this period) and Consumer Price Index (CPI; the new UK government's preferred measure of inflation from 2010); both obtained from the Office for National Statistics (ONS; http://www.statistics.gov.uk/statbase/tsdtables1.asp?vlnk=mm23). Examination of both real data series showed few differences and for simplicity we present just the CPI adjustment. Trends in the volume of cigarette sales by price segment were calculated using GHS (2001–08) and Nielsen (2006–09) data, with the 3-year overlap period from 2006 to 2008 used to ensure comparability.

The tax paid per pack of cigarettes was calculated based on the weighted average retail selling price within each price segment. Average net-of-tax revenue per pack of cigarettes for each price segment was then calculated by subtracting the tax [excise plus value added tax (VAT)] paid from the weighted average price. Results are presented graphically in real terms using the UK CPI.

#### Exploring industry pricing strategy

To explore the extent to which tobacco companies shift tobacco tax increases onto smokers changes in price *net* of tax (deflated using CPI with 2010 as the baseline year) were examined between November 2006 and 2009 by price segment. The price trends by segment were first examined for each of the 3 years. Then, each year was broken into two periods in order to compare average price trends in the periods when duty increases (November–May; duty increases occurring in March or April each year) with the period when duties do not increase (May–November).

Scatterplot graphs were used to explore the relationship between price and market share. The first—a plot of the increase in real prices between 2006 and 2009 against market share in November 2006 for each brand—tested the hypothesis that brands with higher market shares would have larger increases in price. The second—a plot of change in market share over the period 2006–09 against changes in real price—explored whether cheaper brands had larger increases in market share.

#### Comparison of price indices

The two most commonly used price indices for cigarettes during the study period, the MPPC and the RPI for cigarettes, were compared with weighted average prices over the period 2001–09.

## Results

### Trends in cigarette prices, volumes and revenue by price segment

**Figure 1:**
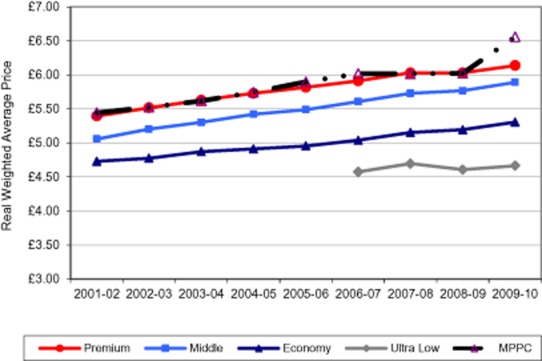
Real weighted average cigarette prices by price segment (2001–2 to 2009–10) using Consumer Price Index (CPI) indexation Source: PriceChecker and General Household Survey (for 2001–05), Nielsen (for 2006–09). CPI data used are Office for National Statistics' (ONS)' ‘all items’ CPI (designation: D7BT). The most popular price category (MPPC) data are published by the Tobacco Manufacturer's Association (TMA) (http://www.the-tma.org.uk/tma-publications-research/facts-figures/uk-cigarette-prices/). The weighted average price measure is based on our own calculations. Note: CPI and weighted average price data are taken from November each year, while the MPPC data are taken form January of the following year. Hence, the year categories have been labelled ‘2001–02’, ‘2002–03’, etc. in this graph.

While the real weighted average price of premium, mid-price and economy brands has increased gradually between 2001 and 2009 (Fig. 1), the real price of ULP cigarettes has barely changed. Consequently, the real price gap between these segments has widened.

**Figure 2:**
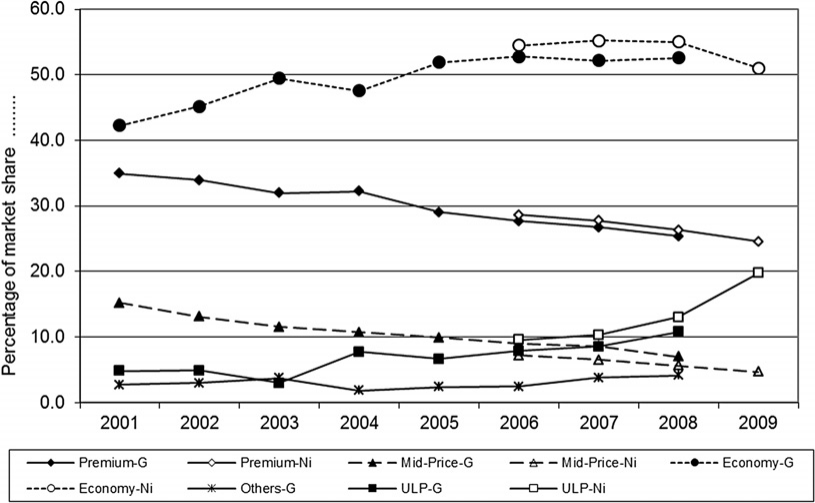
Volume market share by price segment, 2001–09 Source: General Household Survey (GHS) and Nielsen data. G = GHS data; Ni = Nielsen data; ULP = ultra-low price. ‘Others-G’ = market share held by brands that were not identified in the GHS (this includes ‘brand not found’, i.e. the smoker names the brand, but it is not identified in the GHS list; ‘smokes two brands equally’ or ‘no regular brand'—for the last two categories the survey does not attempt to record a brand) or that were identified in the GHS, but for which we were unable to identify price data and thus to allocate to a price segment; the brands in this second group all had very low market shares. Further details are provided elsewhere 11.

Volume data comparisons show that the two data sources (GHS and Nielson) are consistent and provide a reasonable assessment of volume trends over time (Fig. 2). The market share held by ULP cigarettes has increased markedly in recent years, doubling between 2006 and 2009 alone. The largest market share, around 50% of the market, is still held by the economy segment, although this has fallen recently owing to gains in the ULP segment. The market share of both premium and mid-price brands has fallen from 2001 onwards, although mid-price brands are far fewer in number and have never been a major part of the market.

**Figure 3:**
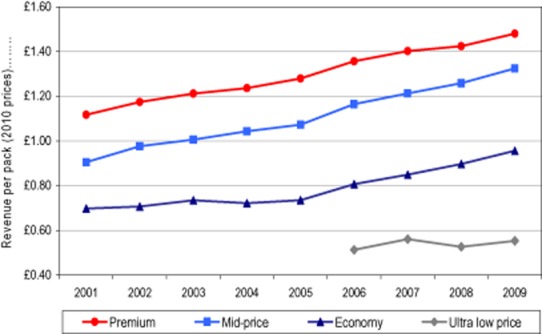
Real (net-of-tax) revenue per pack of cigarettes by price segment, using Consumer Price Index (CPI) indexation, 2001–09 Source: Our calculations using cigarette price data from *PriceChecker* (2001–05) and Nielsen (2006–09), and data on tobacco duty rates 59. CPI data used are Office for National Statistics' (ONS)' ‘all items’ CPI (designation: D7BT).

Consistent with the price data, there is a wide and increasing gap in revenue between premium and ULP brands (Fig. 3). Revenue from premium and mid-price brands has increased in real terms, while revenue from ULP brands has not and the gap in average revenue between these segments now stands at about £1.00. An interesting pattern occurs with economy brands; their revenue is virtually static between 2001 and 2005 but, once ULP brands emerge, revenue in the economy segment starts to increase (Fig. 3).

### Industry pricing strategy

Across the market as a whole, the industry is increasing prices over and beyond tax increases (i.e. overshifting taxes) (Table 1). However, the extent to which tax increases are shifted to smokers varies both over time and by brand segment. Taxes were overshifted in each of the 3 years examined and in all brand segments other than the ULP segment in 2007–08 when taxes were absorbed. Across the 3-year period as a whole, average prices of ULP brands increased by just 1.3 pence compared with between 4.1 and 4.9 pence on more expensive brands. If this 3-year analysis is limited to brands with a market share over 0.2% (data not shown), price falls of −1.2 pence are seen on ULP brands (owing to taxes being absorbed) and price increases of between 4.0 and 5.0 pence in the other segments. The time period in which industry-initiated price changes occur also varies by brand segment. For premium and mid-price segments, price increases during the November–May period (when taxes rise) are greater than for the May–November period. For the economy segment the price increases are more evenly balanced between the two halves of the year. For the ULP segment the pattern is quite different. Net prices *fall* by 3.00 pence during the November to May period (i.e. taxes are undershifted at this point), but increase in the second half of the year.

**Table 1:** Real price increases (net of tax) by brand segment each year and for the periods November–May, May–November and November–November for the 3 years combined (2006–09)

	Real price increases (net of tax) in pence each year			Average real price increase (net of tax) in pence per pack of cigarettes over the 3-year period 2006–9		
		
Price segment	2006–07	2007–08	2008–09	November–May^a^	May–November	November–November
Premium	4.6	2.2	5.7	2.9	1.2	4.1
Mid	5.0	2.7	6.2	3.9	0.7	4.6
Economy	3.5	5.8	5.5	2.1	2.8	4.9
Ultra-low	4.8	−3.6	2.8	−3.0	4.3	1.3
Weighted average, all brands	3.5	1.1	1.3	0.9	1.1	2.0

Source: Nielsen data (http://www.nielsen.com/uk/en.html). Real price data adjusted using Consumer Price Index with 2010 as baseline. ^a^The half of the year in which the tobacco excise increase occurs.

**Figure 4:**
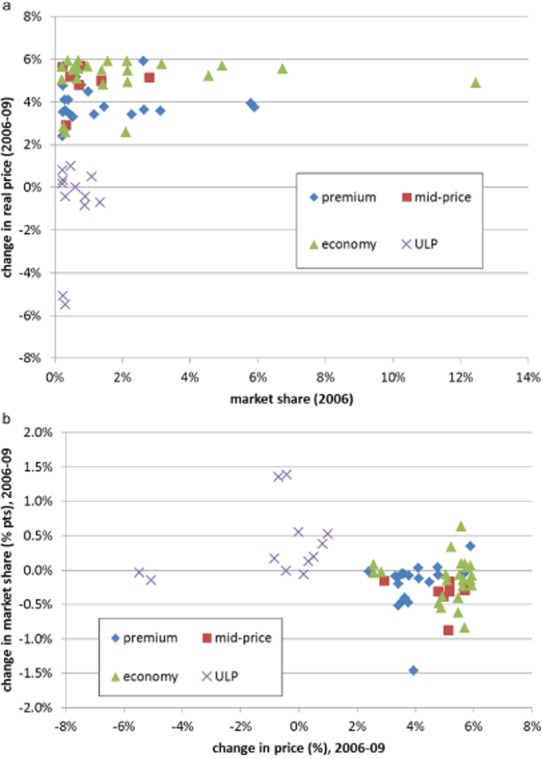
Relationship between price and market share. (a) Change in real price between 2006 and 2009 as a function of market share in 2006 (source: Nielsen data, http://www.nielsen.com/uk/en.html). (b) Change in market share between 2006 and 2009 as a function of change price between 2006 and 2009 (source: Nielsen data). ULP = ultra-low price.

There is little relationship between market share and price increases overall or within each price segment and thus little evidence to support the hypothesis that price increases are greater for brands with larger market shares (Fig. 4a). Consistent with the data in Table 1, brands in the ULP segment have much smaller real price increases than brands in other segments—less than a 1% increase for every brand in this group, with the majority showing no change or *falls* in real price (some as high as −5%). The real price increases for premium brands are lower (mostly between 3% and 5%) than for medium-priced or economy brands (mostly between 5% and 6%).

Overall (and with the exception of a few outliers), change in market share was inversely related to changes in price (Fig. 4b) and ULP brands had both the largest declines in price and the greatest increase in market share.

### Comparison of price indices

**Figure 5:**
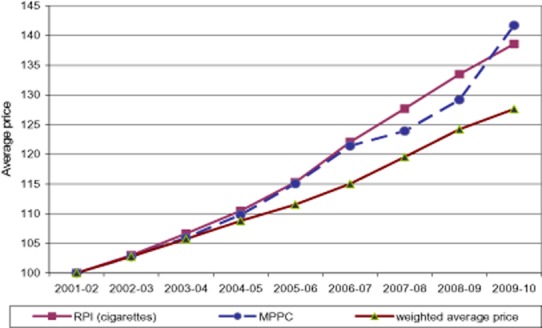
Growth in cigarette price indices, 2001–09 (2001 = 100) Notes: The Retail Price Index (RPI) (cigarettes) measure used here is the Office for National Statistics' (ONS)' ‘cigarettes and tobacco’ Retail Prices measure (designation: CHBE). Source: The most popular price category (MPPC) data are published by the Tobacco Manufacturer's Association (http://www.the-tma.org.uk/tma-publications-research/facts-figures/uk-cigarette-prices/). The weighted average price measure is based on our own calculations. RPI and weighted average price data are taken from November each year while the MPPC data are taken from January of the following year because the MPPC on 1 January each year is based on price data from the end of the previous year (Frank van Driessche, European Commission, 2010, personal communication), and this, therefore, provides the best comparison. Hence, the year categories have been labelled ‘2001–02’, ‘2002–03’, etc. in this graph. Consequently, however, changes to the value added tax (VAT) regime from December 2008 onwards have an effect on the MPPC figures before this effec appears in the RPI and weighted average price (WAP) figures. (Having consistently been 17.5%, VAT reduced to 15% in December 2008, returned to 17.5% in January 2010 and increased to 20% in January 2011.)

The government's RPI measure for cigarettes and the MPPC measure give an exaggerated picture of cigarette price increases (Fig. 5). From 2001–02 to 2009–10, weighted average cigarette prices increased by around 28% compared with 41% for MPPC and 38% for RPI. The MPPC measure tracked the cigarettes' RPI closely up to 2006–07 and the minor subsequent differences are likely being explained by changes to the VAT regime from 2008 onwards differentially impacting the MPPC and RPI data (for full explanation see Fig. 5 footnote).

A comparison of both RPI and MPPC with segment specific prices indicates that both measures almost exactly reflect the price of premium brands and do not, therefore, reflect the growth in volume and smaller price increases of the cheaper price segments (Fig. 1 shows this comparison for MPPC).

## Discussion

### Key findings

Using an extensive literature review and analysis of survey and commercial data we provide, to our knowledge, the most in-depth analysis of tobacco industry pricing strategies yet undertaken. We show that the industry categorises its brands into four price segments from premium to ULP, the latter sold by TTCs only since 2006 whence the number of ULP brands has increased and their market share doubled. The price and revenue gap between the most and least expensive brands has increased since 2001, this gap becoming more marked since ULP brands were introduced in 2006, as their real price has remained virtually static. Our examination of the industry's pricing strategy indicates that this growing gap in price (and revenue) can be explained by the extent to which the industry differentially overshifts taxes between price segments. Between 2006 and 2009, while the industry overshifted taxes by an average of more than 4.00 pence per annum on all other brand segments, it did so by an average of only 1.3 pence on ULP brands. Moreover, at the point when tobacco taxes are increased in March/April each year, the industry overshifts the tax increase on the more expensive brands, while absorbing the tax increase on ULP brands. This suggests that industry-initiated price changes are timed to reassure price-sensitive smokers and accentuate the price gap, but also to hide the price increases on the more expensive brands behind the excise increases.

When examining trends in the real prices of individual ULP brands, we find that some have fallen by as much as 5% and none have increased by more than 1%, while price increases in other brands vary from over 2% to approximately 6%. Unsurprisingly, we find that changes in market share are related to price changes and thus the largest increases in market share are seen in ULP brands.

Although the overshifting of taxes should benefit public health, the opportunities and incentives for consumers to downtrade from expensive to cheaper cigarettes have clearly increased over time and, if current pricing strategy continues, will increase further. Recent research showing that the availability of low cost cigarettes in a market reduces the effect of cigarette price increases in promoting smoking cessation 24 suggests this pricing strategy may undermine the public health impact of tax increases.

Our findings also indicate that the way in which tobacco prices have been tracked, and thus the effect of tobacco tax policies monitored (using cigarettes' RPI or MPPC), has been inadequate. Because both measures simply reflect the price of premium brands, they give an exaggerated picture of cigarette price increases. This helps explain why the problems revealed in this article have not been identified previously and highlights the need for greater scrutiny of tobacco prices, including trends in the four price segments, than has hitherto been the norm.

The TTCs' own documents released via litigation provide insights into why the industry keeps some cigarettes so cheap and who they target with such brands. Existing document research indicates that low-priced brands and price promotions are developed to target young smokers 25, based on the industry's awareness of evidence of the higher price sensitivity of this group and the particular effect of price on the decision to smoke 26,27. Other documents indicate that cheap products, including cigarettes 28 and roll-your-own, have a ‘a tactical role in keeping smokers in the market place when cigarette prices rise’ 28,29—the anticipation being that such smokers will, at some stage, trade-up to more expensive products 30. Such motives are consistent with the identified purposes of cigarette marketing, which include attracting starters, keeping smokers in the market, restarting quitters and boosting consumption 31. TTC documents would thus suggest that ULP brands may perform two functions: keeping price-sensitive smokers in the market and enabling the price-sensitive young to take up smoking.

### Strengths, weaknesses and related evidence

The main weakness of the analyses presented here is that they rely on different data sources over time. This was, however, unavoidable and extensive efforts were made to validate and check consistency of the data over time 11.

The main strength of this work is that it is the first time such price data have been examined in detail in Europe. As such, the work addresses a major research gap identified in a recent review 6. Despite the work's novelty, the findings are consistent with observations in financial analyst and market reports of industry pricing power and the greater profitability of high-end brands 32–35, with empirical evidence of the overshifting of taxes in the USA and other concentrated markets in recent years 6,36–40, and of the importance of price in determining the market share of discount brands in the USA 41. Although one study covering 12 EU countries found undershifting of taxes in the early 1990s 42, there has since been considerable market consolidation in Europe 34,43.

### Implications for policy and practice

Given the importance of price as a tobacco control policy both in encouraging quitting and discouraging uptake, particularly among price-sensitive poorer and younger smokers 5,44 and evidence that price increases are the best intervention to reduce inequalities in smoking 7, this article has important implications for policy.

Despite its documented involvement in cigarette smuggling 45–50, the tobacco industry consistently argues that tax and price increases lead to smuggling 6,51. Yet, we show that, even in the UK, where taxes are among the highest in Europe and globally 52–54, the industry is overshifting taxes, in direct contradiction to its lobbying stance. This suggests the industry is not opposed to *price* increases *per se*, but wants this to occur via its *own* price increases and not via tax increases to ensure that its profits and not government revenues increase. This represents a missed opportunity for governments and highlights the extent to which industry is mis-using the smuggling argument.

The need to narrow the price differential between expensive and cheap cigarettes, and to prevent TTCs from price discounting the cheapest brands is also highlighted. A number of interventions would be effective in this regard. Minimum pricing is one option, but contravenes EU competition rules 6 and could serve to further increase TTC profits, already considerably higher than most consumer staple companies 34. Instead, the latest EU Directive 9 allows a minimum excise tax and we suggest this be set at the highest possible level. Second, maximising the specific element of tobacco excise would narrow the price gap, with specific taxes found to be relatively more effective in increasing prices and reducing cigarette consumption than ad valorem taxes 55. Unfortunately, however, the EU continues to require an ad valorem element 9 and, according to our data, despite the UK having some of the highest levels of specific tax among EU member states 52, in 2009 only 51% of the total tax burden (including VAT) on premium and 58% on ULP brands was specific (our own calculation).

Given evidence of a growth in price-based cigarette marketing 56,57 with at least one campaign selling ULP brands at a loss 58, we suggest that such efforts be combined with a prohibition on below-cost selling and price-based marketing. As the latter sometimes takes the form of price-marked packs 57, this would be facilitated by plain packaging legislation. In the interim, marketing expenditure data (similar to that required by the US Food and Drug Administration) 6 is needed to enable tracking of the industry's use of price-based promotions.

Another option would be to consider price cap regulation 34 in which a cap is placed on the pre-tax cigarette manufacturers' price (and thus on the profit the tobacco companies can make), but not on the retail price that consumers face (i.e. prices will remain high for consumers to deter use). Well established in the utilities industry, price cap regulation would set a maximum price that cigarette companies can charge for their product. Such a system would have the added benefits of addressing the problem of market failure and excess profits in the tobacco industry 34, and of increasing government revenue by transferring the excess profits from the industry to the government purse. It could also help control other unwanted industry practices, such as cigarette smuggling, price-fixing and marketing to the young.

Using weighted average cigarette prices will give more accurate assessment of price trends than RPI or MPPC. The shift from basing tobacco excise rates on the MPPC to the weighted average retail selling price in the latest EU Directive 9 is therefore to be welcomed. However, it is also essential to monitor trends by price segment. Obtaining such data can be prohibitively expensive. Governments should therefore require industry to provide brand- and category-specific price data on a timely basis and, in turn, make such data available to researchers so that tobacco prices can be more closely monitored.

Finally, we note that this research was prompted by one of our observations of rising industry profits combined with the growing market share of cheap cigarettes, highlighting the important role that research on corporations, including monitoring of corporate practices, can play in developing effective public health policy.

### Declaration of interest

This work was funded by EC FP7 Grant Agreement HEALTH-F2-2009-223323, ‘Pricing Policies and Control of Tobacco in Europe (PPACTE)’. AG is supported by a Health Foundation Clinician Scientist Fellowship. AG is a member of the UK Centre for Tobacco Control Studies (UKCTCS), a UK Centre for Public Health Excellence. Funding to UKCTCS from the British Heart Foundation, Cancer Research UK, the Economic and Social Research Council, the Medical Research Council and the National Institute of Health Research, under the auspices of the UK Clinical Research Collaboration, is gratefully acknowledged. The funders played no role in the study design, analysis, and interpretation of data, nor writing of the report or the decision to submit the article for publication.
